# Understanding the Temporal Dynamics of Accelerated Brain Aging and Resilient Brain Aging: Insights from Discriminative Event-Based Analysis of UK Biobank Data

**DOI:** 10.3390/bioengineering11070647

**Published:** 2024-06-25

**Authors:** Lan Lin, Yutong Wu, Lingyu Liu, Shen Sun, Shuicai Wu

**Affiliations:** 1 Department of Biomedical Engineering, College of Chemistry and Life Science, Beijing University of Technology, Beijing 100124, China; wyt191026@emails.bjut.edu.cn (Y.W.); liulingy@emails.bjut.edu.cn (L.L.); wushuicai@bjut.edu.cn (S.W.); 2 Intelligent Physiological Measurement and Clinical Translation, Beijing International Base for Scientific and Technological Cooperation, Beijing University of Technology, Beijing 100124, China

**Keywords:** accelerated brain aging, resilient brain aging, discriminative event-based analysis, brain age prediction, multimodal neuroimaging, UK biobank

## Abstract

The intricate dynamics of brain aging, especially the neurodegenerative mechanisms driving accelerated (ABA) and resilient brain aging (RBA), are pivotal in neuroscience. Understanding the temporal dynamics of these phenotypes is crucial for identifying vulnerabilities to cognitive decline and neurodegenerative diseases. Currently, there is a lack of comprehensive understanding of the temporal dynamics and neuroimaging biomarkers linked to ABA and RBA. This study addressed this gap by utilizing a large-scale UK Biobank (UKB) cohort, with the aim to elucidate brain aging heterogeneity and establish the foundation for targeted interventions. Employing Lasso regression on multimodal neuroimaging data, structural MRI (sMRI), diffusion MRI (dMRI), and resting-state functional MRI (rsfMRI), we predicted the brain age and classified individuals into ABA and RBA cohorts. Our findings identified 1949 subjects (6.2%) as representative of the ABA subpopulation and 3203 subjects (10.1%) as representative of the RBA subpopulation. Additionally, the Discriminative Event-Based Model (DEBM) was applied to estimate the sequence of biomarker changes across aging trajectories. Our analysis unveiled distinct central ordering patterns between the ABA and RBA cohorts, with profound implications for understanding cognitive decline and vulnerability to neurodegenerative disorders. Specifically, the ABA cohort exhibited early degeneration in four functional networks and two cognitive domains, with cortical thinning initially observed in the right hemisphere, followed by the temporal lobe. In contrast, the RBA cohort demonstrated initial degeneration in the three functional networks, with cortical thinning predominantly in the left hemisphere and white matter microstructural degeneration occurring at more advanced stages. The detailed aging progression timeline constructed through our DEBM analysis positioned subjects according to their estimated stage of aging, offering a nuanced view of the aging brain’s alterations. This study holds promise for the development of targeted interventions aimed at mitigating age-related cognitive decline.

## 1. Introduction

Brain aging, also referred to as senescence of the central nervous system, encompasses a gradual decline in cognitive functions and structural integrity [1]. This complex and multifaceted phenomenon is characterized by the progressive loss of neuronal connections, decreased synaptic plasticity, and a diminished capacity for repair and regeneration. As we age, the brain undergoes significant alterations, including volumetric reductions in gray and white matter regions [2], changes in cortical thickness [3], and disruptions in functional connectivity patterns [4]. These alterations not only affect the physical structure of the brain but also its functional capabilities. Understanding the dynamics of both imaging and non-imaging biomarkers during the aging process is crucial. These biomarkers provide critical insights into the changes occurring in the aging brain. By tracking these biomarkers, researchers can elucidate the trajectory of brain aging, identify early signs of cognitive decline, and develop potential interventions to mitigate age-related impairments.

In the realm of neuroscientific research, brain aging in cognitively normal individuals has been meticulously classified to include resilient brain aging (RBA) [5,6,7], normal brain aging, and accelerated brain aging (ABA) [5,8,9], sometimes colloquially termed advanced brain aging. RBA is a concept that describes the ability of certain individuals to maintain neural integrity relatively well as they age. Researchers believe that RBA may be influenced by a multifaceted interplay of factors, including genetic predispositions, a healthy lifestyle, and engagement in mentally stimulating activities. Understanding RBA is crucial as it provides insights into protective mechanisms that could potentially be targeted for interventions aimed at promoting healthy cognitive aging across populations. In contrast, ABA refers to a condition where the brain experiences more pronounced structural and functional changes than those typically observed in peers of the same age group. ABA has been associated with several factors, including chronic stress, poor health, exposure to environmental toxins, and genetic predispositions. Considerable documentation exists regarding distinctive brain aging biomarkers associated with ABA or RBA [10]. Developed in response to the need for more precise tools in neuroscience, Brain Age Gap Estimation (BrainAGE) [11] is an emerging method that quantifies the discrepancy between an individual’s chronological age and their brain’s biological age, as inferred from neuroimaging data. The development of BrainAGE was driven by the recognition that traditional metrics of aging do not capture the complex and heterogeneous nature of brain aging. The estimation of BrainAGE is particularly instrumental in distinguishing between different patterns of brain aging. In the context of RBA, BrainAGE can reveal individuals whose brains exhibit a youthful phenotype relative to their chronological age, indicating a higher level of cognitive and neural resilience [12]. These individuals may possess a more efficient cognitive reserve and better neural maintenance [13]. Conversely, ABA is characterized by a larger-than-expected BrainAGE, where the brain shows signs of aging that exceed the individual’s chronological age. This can be indicative of early or more severe neurodegenerative processes and may be associated with an increased risk of developing cognitive impairments or dementia [13,14,15,16]. The underlying mechanisms of ABA and RBA are complex and remain incompletely elucidated. Therefore, estimating the aging progression timeline in ABA or RBA is crucial for understanding the underlying physiological processes associated with these conditions.

There has been a growing interest in employing data-driven methodologies to explore the dynamics of imaging biomarkers across various stages of aging or disease progression [17]. Among these methodologies, longitudinal approaches stand out as pivotal for offering a temporal perspective on the subtle changes occurring within the brain over extended periods. For example, Jedynak et al. [18] demonstrated the efficacy of longitudinal approaches by reconstructing long-term biomarker trajectories using short-term data. Similarly, Donohue et al. [19] employed a self-modeling regression method to estimate these trajectories, while Sabuncu et al. [20] utilized Cox regression for similar purposes. Despite the methodological straightforwardness of longitudinal approaches, they are often constrained by the stringent requirements of longitudinal data collection. Gathering such data necessitates robust study designs that account for multiple time points, which can be challenging due to issues like participant dropout, variations in imaging data acquisition, and the need for standardized protocols across different time points. In response to these challenges, alternative methodologies have emerged to leverage cross-sectional data to infer the temporal sequence of biomarker abnormalities. One notable method is the Event-Based Model (EBM) [21], which provides a potent probabilistic framework tailored to model disease progression dynamics with a specific focus on biomarkers. The EBM operates by representing changes in a biomarker’s status as discrete “events,” delineating the transition from a “normal” to an “abnormal” state. These events, denoted as *Ei* and arranged in a sequence *S*, are accompanied by the corresponding biomarker set *X*, thereby comprehensively encapsulating the progression trajectory. While the original EBM offered a robust probabilistic framework, a key limitation lies in its assumption that all subjects adhere to a singular sequence of events. To address this limitation, researchers have proposed several modifications to the EBM, enhancing its applicability and robustness. For instance, Young et al. introduced two significant modifications [22,23] aimed at refining the EBM approach. The first modification involves utilizing a dual normal distributions method to independently model each biomarker, thereby offering a more nuanced depiction of biomarker dynamics aligned with real-world data. The second modification relaxes the EBM assumption of a uniform event sequence between subjects, acknowledging the inherent variability in disease progression trajectories. These modified EBMs, along with the original formulation, belong to the generative models family focused on maximizing the likelihood P(X|S). In contrast, discriminative models, such as the discriminative EBM (DEBM) [24], estimate the sequence of events for each subject based on the posterior probability of individual biomarkers transitioning to an abnormal state. The adoption of discriminative models, like the DEBM, has demonstrated improved handling of large datasets and enhanced the stability of abnormal event ordering, thereby contributing to more accurate prognostic and diagnostic assessments in neuroimaging research.

Throughout this scientific inquiry, Lasso regression was employed to predict brain ages using features extracted from structural magnetic resonance imaging (MRI) (sMRI), diffusion MRI (dMRI), and resting-state functional MRI (rsfMRI). These feature sets included 207, 144, and 210 features from sMRI, dMRI, and rsfMRI, respectively. By leveraging these multimodal imaging modalities, Lasso regression offered a comprehensive approach to delineating brain aging trajectories. BrainAGE played a crucial role in stratifying the aging population into ABA and RBA categories. Within our dataset, 1949 subjects were representative of the ABA subpopulation, and 3203 subjects were representative of the ABA subpopulation. ABA or RBA subjects were further categorized into middle-old-aged and old-aged groups based on their specific age ranges. To track the progression of aging events, the DEBM was employed. Initially, the DEBM approximated the event sequence for each subject. Subsequently, a generalized Mallows model was applied to these approximate subject-specific event sequences, leveraging the probabilistic Kendall’s tau distance to derive a central event sequence that encompassed all subjects. This relative distance between events facilitated the construction of an aging progression timeline, positioning subjects according to their estimated stage of aging progression. Through a meticulous dissection of these multimodal MRI features, our investigation aimed to yield novel insights into the complex landscape of ABA and RBA, thereby advancing our comprehension of the underlying mechanisms inherent in the aging process.

The structure of the subsequent sections is outlined as follows: Section 2 provides an extensive introduction, detailing essential aspects, such as the utilization the UK Biobank (UKB) data, the neuroimaging processing pipeline, the machine learning model applied for the brain age prediction, the estimation of the ABA and RBA aging progression timelines, and a thorough exposition of the statistical methodologies foundational to this study. Section 3 presents the study findings in meticulous detail. Subsequently, Section 4 conducts an in-depth discussion situating the findings within the broader landscape of neuroimaging research and the understanding of ABA and RBA. Finally, Section 5 encapsulates a concise summary of the study.

## 2. Materials and Methods

### 2.1. Participants

The dataset utilized in this study was derived from the UKB, which is a large-scale prospective study that enrolled around 500,000 participants aged 37 to 73 years between 2006 and 2010 [25]. The UKB, which is accessible online at www.ukbiobank.ac.uk (accessed on 12 March 2022), obtained ethical approval from the North West Multicentre Research Ethics Committee, with a specific reference number of 11/NW/0382. Additionally, the research presented in this study received authorization from the UKB, with the designated application number being 68,382.

A subset of UKB participants underwent neuroimaging examinations. All brain imaging data were collected utilizing a 3 T Siemens Skyra scanner Siemens Healthcare GmbH, Erlangen, Germany). T1-weighted MRI scans were performed using a magnetization-prepared rapid gradient-echo (MPRAGE) sequence, yielding images with a 1 × 1 × 1 mm voxel size; a 208 × 256 × 256 mm^3^ image matrix; and inversion time (TI) and repetition time (TR) parameters set at 880 and 2000 ms, respectively. The DMRI utilized two b-values, achieving a spatial resolution of 2 × 2 × 2 mm and sampling 100 unique diffusion directions. The rsfMRI sessions were conducted with parameters yielding a spatial resolution of 2.4 × 2.4 × 2.4 mm, a TR of 0.735 s, and a TE of 39 milliseconds. Cognitive evaluation was based on a neuropsychological battery encompassing nine cognitive domains [26]. Notably, two cognitive scales under scrutiny—reaction time (UKB ID: 20023) and trail-making (UKB ID: 6350)—both measuring time as an outcome, were logarithmically transformed to enhance the analytical robustness.

**Figure 1:**
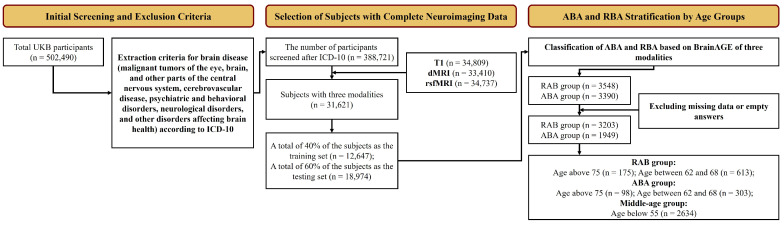
Flowchart illustrating the subject-screening process.

The detailed selection process is detailed in Figure 1. The exclusion criteria were based on the International Classification of Diseases, Tenth Revision (ICD-10) diagnostic classification system. This screening resulted in 388,721 subjects aged 45–83. From this, 31,621 subjects with complete sMRI, dMRI, and rsfMRI data were selected and split into a training set (40%) and a test set (60%) for brain age prediction. Within the test set, individuals with a consistently positive BrainAGE across all imaging modalities were categorized as ABA, and those with consistently negative values as RBA. Participants lacking complete cognitive test data or covariates were excluded, leaving 3203 in the RBA group (mean age of 63.6 years, standard deviation of 7.97) and 1949 subjects in the ABA group (mean age of 64.6 years and a standard deviation of 6.96). Given the relatively slow pace of biomarker changes in ABA or RBA compared with neurodegenerative diseases, we categorized the study participants into three age groups (middle-aged, middle-old-aged, and old-aged), with a 7-year interval between each group to ensure distinct biomarker trajectories across different age spans. Specifically, the middle-aged group (serving as the reference group) consisted of all participants under the age of 55 in the test set, while the middle-old-aged and old-aged groups were drawn from the ABA and RBA cohorts.

### 2.2. Image Processing

The UKB provides a broad spectrum of neuroimaging modalities [27]. Following this, a standardized methodology is applied for image preprocessing and initial analysis. This meticulous approach results in the creation of a vast collection of imaging-derived phenotypes (IDPs).

#### 2.2.1. T1-Weighted MRI (T1)

T1 MRI is a non-invasive modality crucial for examining the intricate anatomy of the human brain. Volume quantification was carefully performed using the FMRIB software library (FSL, version 5.0.10), which is accessible via the platform (http://fsl.fmrib.ox.ac.uk/fsl, accessed on 12 March 2022). Additionally, the FMRIB’s automated segmentation tool (FAST, version FAST3) was deployed to derive a total of 139 IDPs (ROIs) [28]. The cortical thickness specific to the ROIs was obtained from the FreeSurfer anatomical parcellation [29], utilizing the Desikan–Killiany Atlas [30] for cortical regions, identifying 68 ROIs, with 34 in each hemisphere.

#### 2.2.2. DMRI

DMRI assesses the white matter microstructure by mapping water diffusion patterns. In this investigation, the DTIFIT tool (accessible at https://fsl.fmrib.ox.ac.uk/fsl/fdt, accessed on 16 March 2022) computed the fractional anisotropy (FA) and mean diffusivity (MD). Furthermore, NODDI (Neurite Orientation Dispersion and Density Imaging) estimated the isotropic water volume fraction (ISOVF). Tract-based spatial statistics (TBSS) [31] was employed for the spatial statistics. The FA images were aligned onto a white matter skeleton using FNIRT-based warping [32], then applied to other dMRI measures. The resulting skeletonized images for each dMRI measure were subjected to averaging across a predefined set of 48 standard spatial tract masks, as defined by Susumi Mori’s group at Johns Hopkins University [33]. The resulting images were averaged across 48 standard spatial tract masks, yielding 144 IDPs.

#### 2.2.3. RsfMRI

The analysis of the rsfMRI images employed the MELODIC (Multivariate Exploratory Linear Decomposition into Independent Components) framework [34], integrating group principal component analysis and independent component analysis to extract spatially orthogonal independent components (ICs) that represented distinct resting-state neural networks. A low-dimensional group-independent component analysis approach yielded a population-level spatial map of resting-state networks. Prior to the analysis, functional images underwent preprocessing with 25 fractions (UKB ID: 25752). After a careful exclusion process that eliminated four noise components, 21 components of interest remained, each representing unique resting-state networks. Additionally, a partial correlation matrix derived from rsfMRI data was utilized to quantify the network connections, yielding 210 values.

### 2.3. Brain Age Prediction Model

The Lasso (least absolute shrinkage and selection operator) is a regression technique that enhances linear models by penalizing coefficients to prevent overfitting, aiding generalization and variable selection. Its superiority in brain age prediction is well-documented [34,35]. Hence, we employed the Lasso in our research, with the regularization parameter (alpha) crucial for controlling the penalty magnitude. We meticulously defined the alpha grid search space (0.001, 0.01, 0.1, 1, 10, 100) to optimize model performance via fivefold cross-validation on the training dataset.

The BrainAGE [12] metric compares the estimated age of a person’s brain with their actual chronological age, providing insights into their brain’s aging trajectory compared with peers. This assessment not only sheds light on the overall state of brain maintenance (BM) [10,36] but also provides valuable insights into the presence and extent of any underlying neuroanatomical irregularities. The brain age prediction model is an advanced neuroscientific tool designed to estimate an individual’s brain age through the analysis of neuroimaging data. It utilizes machine learning algorithms to discern patterns and features that differentiate the biological age of the brain from the chronological age. Computing the BrainAGE score involves determining the difference between the age forecasted by the model and the individual’s chronological age (Equation (1)). A score close to zero suggests typical aging, while positive scores indicate accelerated aging, and negative scores suggest a younger brain.

BrainAGE = Predicted brain age − Chronological age(1)

To mitigate age-related bias, an age-bias correction procedure (Equation (2)) is crucial.

Corrected brain age = Predicted brain age − α − β × Chronological age(2)

**Figure 2:**
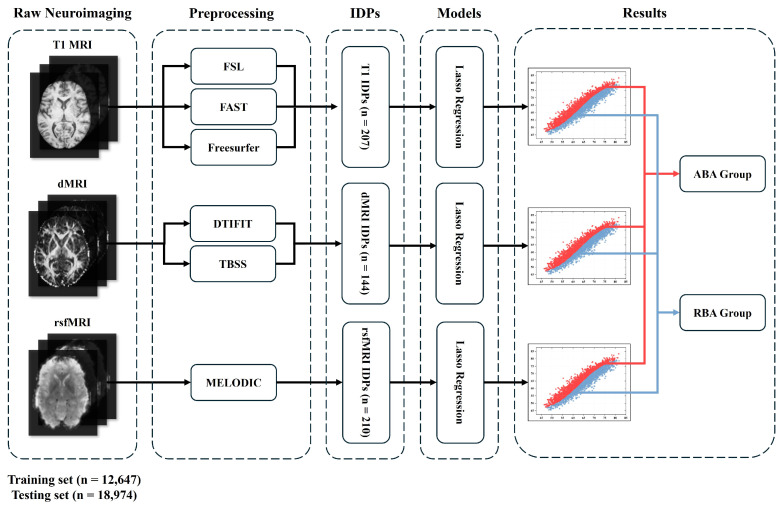
Flowchart illustrating the categorization of ABA and RBA subjects.

Here, “predicted brain age” refers to the brain age estimated by the Lasso model, while α and β represent the intercept and slope of the regression line from the chronological age and predicted age. Subjects in the test sets were categorized based on BrainAGE. Those with positive BrainAGE values across all three modalities were allocated to the ABA group, indicating accelerated brain aging. Conversely, individuals exhibiting negative BrainAGE values across the three imaging modalities were assigned to the RBA group, suggesting a more youthful brain state compared with their age. The detailed flowchart figure was shown in the Figure 2.

### 2.4. Estimating Biomarker Ordering Using DEBM

Utilizing cross-sectional data, the EBM characterizes the structured advancement of cumulative degenerative processes associated with aging, while concurrently assessing the inherent uncertainty in this progression. Our study employed the DEBM, which is a well-established framework accessible at https://github.com/EuroPOND/pyebm (accessed on 8 August 2023). The DEBM constitutes a category of progression modeling that leverages cross-sectional data to predict the most likely sequential occurrence of events, focusing on biomarker degeneration during aging. Individuals are then assigned an aging stage based on their biomarker values within this sequence. The DEBM has been employed in a spectrum of neurological disorders, encompassing Alzheimer’s disease (AD) [24,37], Parkinson’s disease [38], amyotrophic lateral sclerosis [39], and multiple sclerosis [40]. Extensive validation studies highlighted the DEBM’s superior accuracy and computational efficiency compared with other EBM implementations [24,41]. The DEBM functions by utilizing cross-sectional data to infer the most probable sequence of events, particularly the degradation of biomarkers, which serve as indicators of the aging process. After evaluating their biomarker values, each individual is subsequently categorized into an aging stage within a sequence. This classification process is founded upon a probabilistic framework, wherein each biomarker undergoes evaluation to determine its status as either normal or degenerated, with the transition between these states regarded as a pivotal event. The main goal is to reveal the most likely ordered cascade of these events, outlining an individuals’ trajectory from a state of health to the wide spectrum of manifestations associated with aging.

**Figure 3:**
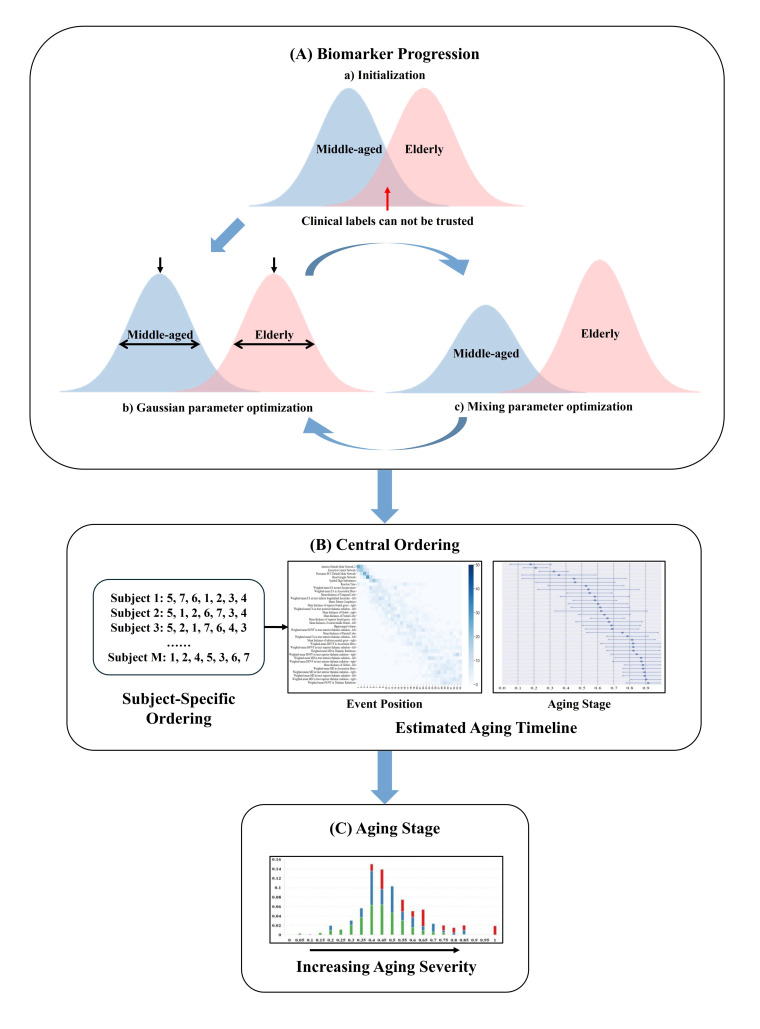
Overview of DEBM steps: (**A**) Biomarkers are transformed into probabilities of degeneration using GMM to estimate normal and degenerated states, as classified via Bayesian methods. (**B**) Subject-specific biomarker degeneration sequences are inferred and used to derive a central ordering for aging progression timelines. (**C**) The central ordering stages subjects based on aging severity.

Figure 3 depicts the procedural steps of the DEBM. Initially, the model calculates a subject-specific sequence based on the posterior probability of biomarker degeneration. This sequence is personalized, derived from the individual’s biomarker profile, indicating a unique progression of degenerative events. Next, the central sequence, regarded as the population’s most typical event order, is determined. This sequence minimizes the aggregate of the probabilistic Kendall’s tau distances in comparison with all subject-specific sequences. The DEBM acknowledges that individual subject sequences serve as noisy approximations of the central sequence, owing to physiological variations in biomarker expression. The DEBM employs a specialized mixture model. This model is initiated by estimating the distributions of biomarker values for middle-aged and elderly (ABA or RBA) subjects, leveraging data from individuals spanning the aging spectrum’s extremes. A Bayesian classifier is trained to discern and exclude outliers and potentially mislabeled data. This method effectively segregates Gaussian distributions representing the normal and degenerated states of each biomarker. The initially biased distributions are then refined by integrating a broader dataset, encompassing middle-old-aged (ABA or RBA) subjects. This cohort displays a blend of biomarkers in both normal and degenerated states, including instances that may have been previously misclassified. The refinement process is facilitated by a Gaussian mixture model (GMM), which applies constraints derived from the relationship between the expected and biased distributions. The GMM function is iteratively fine-tuned with regard to both Gaussian parameters and mixing parameters until convergence is achieved. Optimal sequences are derived as the averages of orderings obtained from 50 bootstrapped iterations for the DEBM. Employing 10-fold cross-validation, each participant is assigned an aging stage. These delineated aging stages are exclusively derived from individual biomarker profiles and their alignment along the aging progression continuum, as determined by the estimated sequence of biomarker alterations. Importantly, the characterization of these aging stages is independent of the individual’s chronological age, highlighting the model’s ability to deepen our understanding of aging beyond chronological years.

### 2.5. Selected Biomarkers

For enhanced interpretability and computational efficiency, not all multimodal neuroimaging biomarkers could be comprehensively utilized in the DEBM analysis. Therefore, a meticulous selection process was undertaken, which involved drawing from a pool of multimodal neuroimaging biomarkers and cognitive data and resulted in the identification of 34 biomarkers. These selected biomarkers exhibited specific characteristics crucial to the efficacy of our analysis. First, they encompassed a wide range of features, allowing for a holistic representation of the aging process in the ABA. Moreover, these selected biomarkers excelled in distinguishing between normal and degenerated states.

Expanding our analysis beyond these IDPs (local features), we incorporated global features, namely, left and right cortical thickness, alongside mesoscopic scale features. Mesoscopic scale features were determined based on brain lobes or white matter anatomical locations and connectivity functions. The computation of these features relied on the feature values from the included brain regions. For each feature, we further computed the Cohen’s d effect size between the middle-aged and elderly groups, leveraging all acquired multimodal imaging IDPs, global features, and mesoscopic scale features, in conjunction with nine cognitive tests. A Cohen’s d value of 0.8 or higher indicates a large effect size. In instances where Cohen’s d exceeded 0.8, indicating significant differences, biomarker selection was guided by the descending order of Cohen’s d values within each category, reflecting the magnitude of the differences observed. Neuroimaging biomarkers were stratified into three primary classes: global, mesoscopic scale, and brain regions. Global biomarkers encompassed the left and right cortical thicknesses. Mesoscopic scale features retained four each of the gray and white matter features, which totaled eight. The local feature sets included the average cortical thickness, weighted average FA, MD, ISOVF for white matter fibers, and hippocampus volume. Each of the first four local feature sets retained four features: Freesurfer (UKB ID: 26781, 26782, 26863, 26883), FA (UKB ID: 25490, 25499, 25502, 25507), MD (UKB ID: 25517, 25518, 25538, 25539), and ISOVF (UKB ID: 25706, 25707, 25727, 25728), which resulted in a total of 17. Additionally, four nodes of rsfMRI high-order functional networks and three cognitive tests (reaction time, substitution of numerical symbols, and completion of matrix patterns) were included, which resulted in a total selection of 34 features.

## 3. Results

### 3.1. Brain Age Prediction

Within the confines of this investigation, Lasso regression analysis was chosen for forecasting brain ages, with the mean absolute error (MAE) employed as the benchmark to gauge the model effectiveness. Notably, dMRI showed the highest predictive accuracy among the modalities. The implementation of Lasso regression on the dMRI data yielded a low MAE of 4.03 years. Furthermore, the T1 data, including the cortical thickness and gray matter volume, achieved an MAE of 4.17 years, whereas the rsfMRI showed a higher MAE of 5.28 years. The classification of the ABA and RBA groups hinged upon the consistency of positive or negative BrainAGEs across the three modalities within the brain-age prediction test set (*n* = 18,974). Specifically, the subjects were categorized as ABA if the BrainAGE across all modalities was consistently positive; conversely, the subjects were classified as RBA if the BrainAGE consistently exhibited negativity. As a result, 3203 subjects were classified into the RBA group (mean age = 63.6 ± 7.97), and 1949 subjects were classified into the ABA group (mean age = 64.6 ± 6.96).

### 3.2. Sequence of Biomarker Degeneration for ABA and RBA

**Figure 4:**
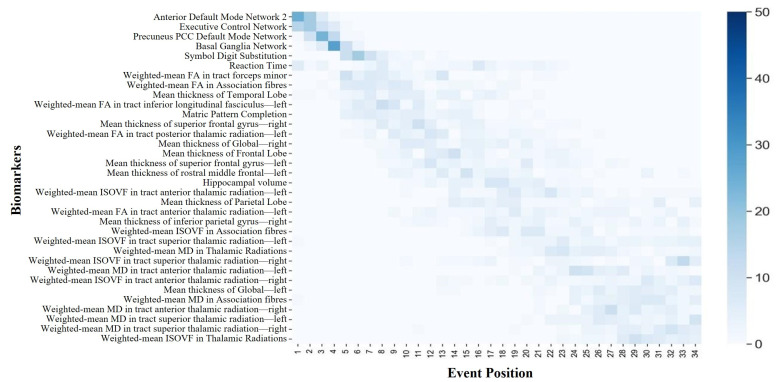
The positional variance diagram illustrating the sequence of biomarker degeneration of the ABA cohort. The *y*-axis (top to bottom) represents the maximum likelihood sequence of biomarker degeneration.

**Figure 5:**
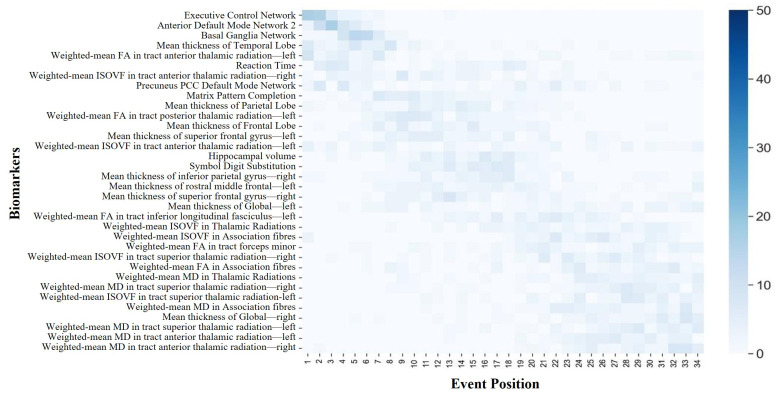
The positional variance diagram illustrating the sequence of biomarker degeneration of the RBA cohort.

The DEBM estimated the sequence for the thirty-four selected biomarkers, assigning them to stages 1 through 34. Figure 4 and Figure 5 illustrate the sequence of biomarker degeneration and corresponding uncertainty in the ABA and RBA cohorts. Here, each square’s color intensity signifies the frequency the bootstrap resampling iterations placed the biomarker at a specific position. Thus, the darkest square for each biomarker indicates its most frequent position, representing the mode. Such representation aids in grasping the biomarker position distribution and insights into the sequence’s mode. For the ABA cohort, four functional networks served as the initial biomarkers to undergo degeneration, followed by two cognitive domains. In terms of the whole-brain cortical thickness, degeneration manifested in the right hemisphere preceding the left hemisphere. Notably, at the mesoscopic scale of cortical thickness, reductions in the temporal lobe cortical thickness preceded those in the frontal lobe. Within the mesoscopic white matter microstructure, degeneration in the FA of association fibers preceded degeneration in the ISOVF in association fibers. At the microscopic brain region level, the FA-related features appeared earlier, the ISOVF-related features appeared later, and the MD-related features exhibited anomalous transitions in the final stages. It is noteworthy that vulnerable regions in neurodegenerative diseases, such as the hippocampus, only demonstrated degeneration in the intermediate stages. In the RBA cohort, it is noteworthy that the biomarker ordering emphasized the executive control network, anterior default network 2, and basal ganglia network as the earliest markers of degeneration. However, unlike the ABA cohort, degeneration in the whole-brain cortical thickness manifested in the left hemisphere preceding the right hemisphere. At the mesoscopic scale of cortical thickness, both groups demonstrated alterations in the temporal and frontal lobes; however, in the RBA cohort, degeneration of the temporal lobe ranked fourth in prominence. Concerning the white matter microstructure, degeneration in the RBA group primarily occurred at relatively advanced stages. Degeneration associated with the MD tended to occur later than other measures of white matter microstructure, with gray matter degeneration preceding white matter degeneration. Furthermore, in the RBA cohort, the biomarker ordering exhibited a higher degree of uncertainty compared with that of the ABA cohort.

**Figure 6:**
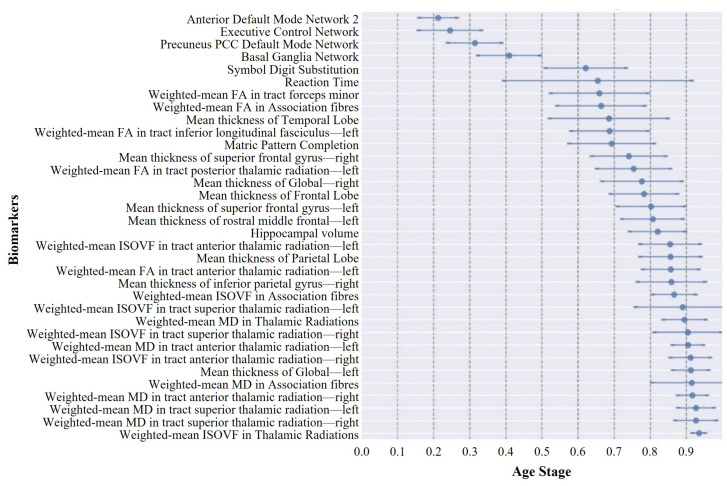
The event-center variance diagram illustrating the standard error of the estimated degeneration centers of the ABA cohort. The plot displays the event-centers of the various biomarkers, along with their respective standard deviations, as estimated from a batch of 50 independent bootstrap samples.

**Figure 7:**
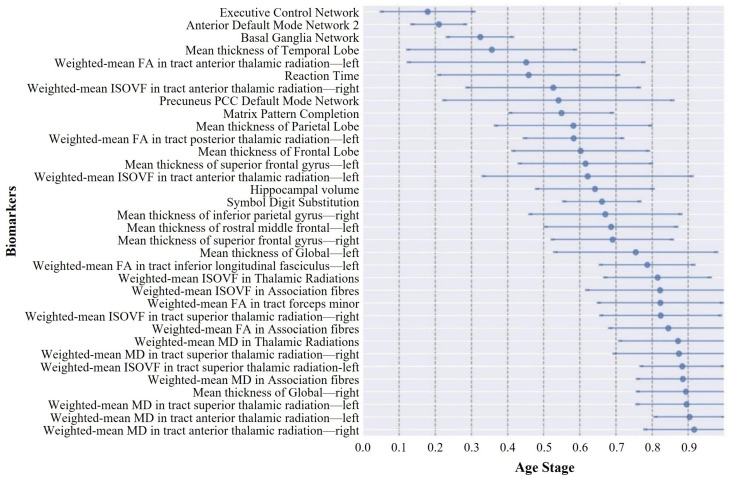
The event-center variance diagram illustrating the standard error of estimated degeneration centers of the RBA cohort.

Figure 6 and Figure 7 display the event center variance plots tailored to the ABA and RBA cohorts. These charts comprehensively delineate the trajectory of biomarker degeneration events over the aging timeline, emphasizing the relative temporal positioning of these events in relation to each other. A notable observation is the distinct divergence in aging trajectories between the ABA and RBA cohorts. In contrast to the RBA cohort, the biomarkers within the ABA cohort generally showed smaller event center standard deviations, suggesting a more consistent and predictable trend of biomarker degeneration. This uniformity could stem from similarities in pathological physiological changes experienced by individuals within the ABA cohort, leading to relatively stable trajectories of biomarker degeneration. In contrast, the larger event center standard deviation of the biomarkers in the RBA cohort indicates more variability in the timing and sequencing of the biomarker events within this cohort. This heterogeneity may reflect the activation of different protective mechanisms by individuals in the RBA cohort, which may delay the progression of cognitive decline, leading to a slower and more diverse aging process.

### 3.3. Estimation of Aging Stage for ABA and RBA

**Figure 8:**
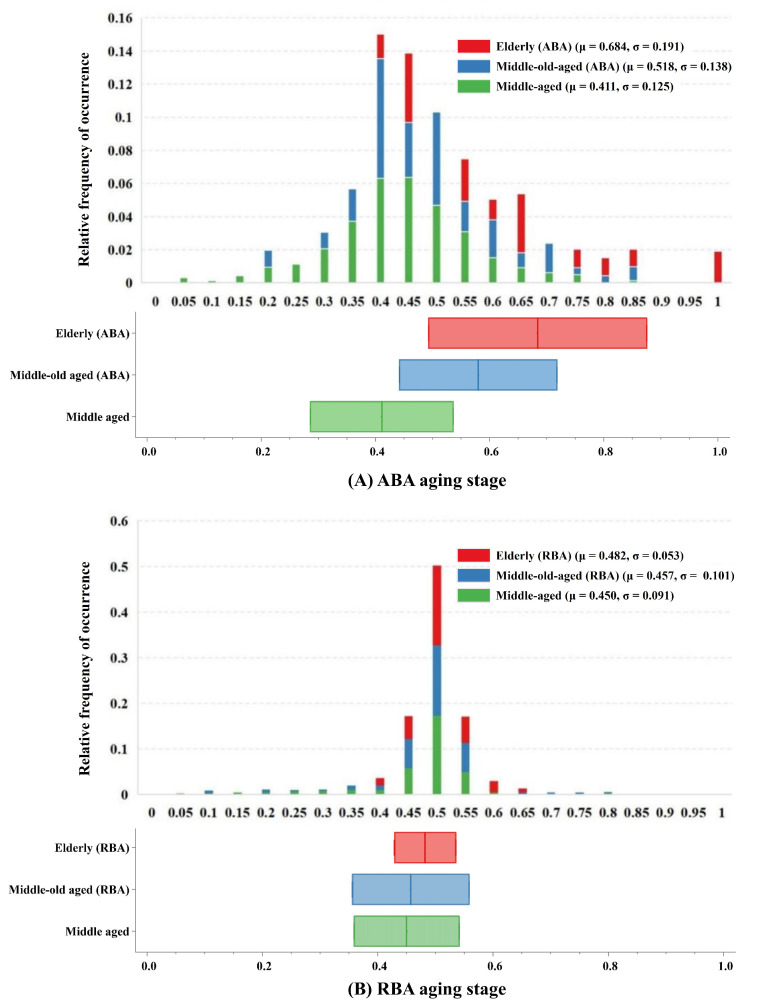
Estimation of aging stages across age groups. The aging stages were estimated utilizing a 10-fold cross-validation methodology. Each histogram illustrates the relative frequency distribution of aging stages within each clinical group, normalized for the respective age cohorts. Box plots displaying estimated aging stages for each cohort. The box plots depict the mean value, along with the standard deviation of the aging stage.

The aging stage, which was a summarizing metric for each subject, was determined by estimating the subject’s progression along the established timeline of aging progression (Figure 8). We employed a 10-fold cross-validation approach. Within each fold of the cross-validation, the DEBM was constructed using the training set, while the estimation of the aging stage was conducted on the test set. Within the ABA cohort, which was known for accelerated brain aging, most individuals within the middle-aged or middle-old-aged (ABA) range were positioned toward the left side, indicating less frequent biomarker degeneration. This suggests a relatively moderate trajectory of neurodegenerative changes in this cohort. Conversely, the elderly population (ABA) was predominantly located toward the right. This distribution suggests a swifter and more pronounced advancement of neurodegenerative processes, consistent with advanced age and increased susceptibility to neurodegenerative diseases. In contrast, the RBA cohort, characterized by its resilience to brain aging, exhibited consistent assignment of low or intermediate aging stages across various age brackets, with minimal variation in aging stages observed between different age groups. The minimal variation in aging stages between different age groups within the RBA cohort suggests that the aging process, especially neurodegeneration, may not manifest as prominently or could progress at a slower rate in this demographic. This phenomenon could stem from genetic, lifestyle, or environmental factors fostering a more resilient aging trajectory, rendering it less prone to the customary neurodegenerative alterations associated with aging. These findings underscore distinct patterns of age-related changes in neural integrity between cohorts, potentially reflecting varied trajectories of neurodegeneration and resilience across the ABA and RBA populations.

In the ABA cohort, the majority of middle-aged or middle-old-aged subjects exhibited intermediate aging stages, with a relatively low incidence of biomarker degeneration. Conversely, elderly subjects were predominantly assigned to later stages of aging, indicative of pronounced neurodegenerative processes with advancing age. In contrast, within the RBA cohort, individuals across different age brackets were uniformly assigned low or intermediate aging stages, while lacking notable variation in aging stages between different age groups. This observation implies that age-related neurodegeneration may not manifest prominently within the RBA population as age advances.

## 4. Discussion

Current knowledge of aging-related degeneration in ABA and RBA cohorts primarily stems from studies that focused on various imaging features. This collective body of research has pinpointed regional atrophy, alterations in functional networks, and changes in white matter tract microstructure as notable features. However, the specific sequence of degeneration among these biomarkers has remained largely elusive. To fill this gap, we conducted a data-driven DEBM analysis in this study. ABA saw an initial decline in four functional networks and two cognitive domains, where cortical thinning started in the right hemisphere and then the temporal lobe, FA changes preceded the ISOVF, and the MD exhibited anomalous transitions. Conversely, RBA showed early degeneration in executive control, default network 2, and basal ganglia networks, with cortical thinning predominantly in the left hemisphere and advanced-stage white matter degeneration. These findings highlight nuanced aging dynamics and complex mechanisms in both cohorts. This analysis shed light on the temporal dynamics of the aging progression of the ABA and RBA cohorts, providing valuable insights into the underlying mechanisms and guiding future research directions.

### 4.1. Central Ordering

The central ordering, resembling the main narrative, depicts the overall sequence of biomarker changes during the aging progression. To establish a central ordering, it is crucial to comprehend the variability in the biomarker changes during the brain-aging process across different subjects. Each individual may manifest a unique sequence of biomarker alterations. To account for these individual variances, a distinct change sequence is initially delineated for each subject, analogous to crafting a personalized timeline for the degradation of their biomarkers. However, the scope of the study transcended individual subjects; it aimed to discern common patterns shared among all the participants. By amalgamating the individual sequences of all the participants, the predominant sequence of changes was identified by employing the generalized Mallows model. This methodology identified the most frequently occurring sequence of biomarker degeneration events through the comparative analysis of biomarker degeneration across each individual. This central sequence served as a representative rendition, akin to an average, aiding in the comprehension of the overarching trajectory of biomarker decline during brain aging. Additionally, the Kendall’s tau distance was utilized in this process to measure the dissimilarities between different sequences, thereby ascertaining the requisite number of exchanges to align two sequences. Through this methodological approach, a more accurate estimation of the general sequence of biomarker changes associated with aging progression could be achieved.

The central ordering analysis revealed heterogeneity in the central ordering of both the ABA and RBA sequences. Early degeneration of functional networks was evident in the ABA sequence. As an individual journeys through the aging process, noticeable changes arise in the activation patterns of individual brain networks. These age-related modifications spur individuals to develop compensatory strategies, utilizing alternative networks to counteract the onset of declining cognitive functions [42,43,44]. This adaptive approach aims to alleviate the impact of age-related cognitive decline throughout the aging process and may manifest before a noticeable decline in cognitive function. However, compensatory mechanisms can only partially offset the decline in cognitive abilities, with further deterioration in cognitive domains occurring as age advances [45,46], affecting crucial functions, such as memory and attention. Hemisphere-specific degeneration in the ABA sequence showed earlier onset in the right hemisphere, which was potentially associated with asymmetrical brain aging [47]. In the realm of adult brains, cortical asymmetry, which is characterized by the unequal thickness of the left and right sides of the cortex, is a notable phenomenon. This asymmetry is not merely incidental; rather, it serves a functional purpose, optimizing brain performance by assigning distinct roles to each hemisphere. Conventionally, the left hemisphere is closely associated with language processing, logical reasoning, and sequential tasks, whereas the right hemisphere excels in spatial perception, facial recognition, and aesthetic appreciation. In the context of aging brains, two prominent models elucidate cortical asymmetry changes: the Right Hemi-Aging Model and the Hemispheric Asymmetry Reduction in Older Adults (HAROLD) model. The Right Hemi-Aging Model posits a heightened vulnerability of the right hemisphere to age-related alterations [48], which is a conjecture supported by various studies [48,49], highlighting a more pronounced change in the right hemisphere relative to its left counterpart. Additionally, the early reduction in temporal lobe cortex thickness aligns with typical pathological features of neurodegenerative diseases like AD, given the pivotal role of the temporal lobe in memory formation. Prior studies have also shown associations between mild cognitive impairment (MCI), AD, and accelerated brain aging [12,13]. In contrast, early degeneration in the RBA sequence highlighted the involvement of executive control networks, anterior default mode network 2, and basal ganglia networks, which are regions closely associated with executive functions, self-reflection, and motor control. This suggests that these functions may be affected earlier in the RBA sequence. Notably, degeneration in the left hemisphere of the RBA sequence preceded that in the right hemisphere. However, concerning event centers, the contrast between the hemispheres was less marked compared with the ABA sequence. This observation could potentially be elucidated through the HAROLD model, which suggests that with advancing age, the functional asymmetry between the left and right hemispheres of the brain gradually diminishes [50,51]. In essence, older adults tend to engage both hemispheres in cognitive tasks that were previously lateralized to one hemisphere in younger adults. This compensatory mechanism is believed to aid older adults in maintaining cognitive performance despite age-related changes in their brain structure and function. RBA highlights the brain’s adaptability and resilience to age-related changes. Models such as brain reserve and cognitive reserve have been employed to explicate RBA [52,53]. Research suggests individuals with higher cognitive reserve show less disparity between hemispheres [54,55]. Furthermore, the degeneration of white matter microstructure in the RBA sequence occurred at later stages, indicating that white matter damage may not be an early marker of aging in this sequence but rather associated with later-stage progression. The comparison of the two sequences also highlighted differences in white matter microstructural changes. Early degeneration of the FA in the ABA sequence may indicate early reorganization of white matter fibers, while late-stage degeneration of the MD in the RBA sequence may be related to severe impairment of white matter integrity in the later stages of aging. Additionally, the higher uncertainty in biomarker ordering in the RBA sequence may reflect greater variability in aging progression, which is possibly influenced by differences in genetic backgrounds, lifestyles, or environmental factors between individuals in the RBA group.

### 4.2. Event Centers

The event center can be regarded as a pivotal moment in the process of brain aging. Throughout the aging trajectory, alterations in biomarkers resemble distinct milestones along the path of aging progression. Across the aging continuum, shifts in biomarkers mirror distinct milestones in the trajectory of aging. Analyzing the temporal dynamics of brain aging and pinpointing the timing of various biomarker changes is like identifying specific chapters in a storybook where each significant event happens. To achieve this, comparing data across subjects yields a plausible sequence of event occurrences, yet merely knowing which events precede others is insufficient. Understanding the relative timing of these events, whether early or late in the aging process, is equally important. Understanding the relative timing of these events, whether early or late in the aging process, is equally important. To identify these time points, the probabilistic Kendall’s tau distance was employed, which computes the distance between events by comparing the sequence of events across different subjects. Essentially, events that frequently co-occur have a shorter distance between them, whereas events that rarely co-occur have a greater distance. Subsequently, by calculating these distances, the “event centers” for each event are derived. Event centers represent the average temporal occurrence points for biomarker changes. Additionally, two hypothetical events—one occurring before disease onset and another after disease cessation—are introduced to help determine the earliest and latest points on the timeline. Through the discernment of these event centers, researchers can garner enhanced insights into the gradual progression of aging within both the ABA and RBA cohorts.

A notable observation was the distinct divergence in aging trajectories between the ABA and RBA cohorts. Compared with the RBA cohort, biomarkers within the ABA cohort typically exhibited smaller event center standard deviations, implying a more uniform and predictable trend of biomarker degeneration within the ABA cohort. This uniformity may be associated with greater similarity in pathological physiological changes experienced by individuals within the ABA cohort, resulting in relatively fixed trajectories of biomarker degeneration. In contrast, the larger event center standard deviation of biomarkers in the RBA cohort suggests greater variability in the timing and sequencing of biomarker events within this cohort. This heterogeneity may reflect the activation of different protective mechanisms [56] by individuals in the RBA cohort, which may delay the progression of cognitive decline, leading to a slower and more diverse aging process.

### 4.3. Aging Stages

Aging stages, which consider the relative distance between events, serve as indicators of an individual’s progression along the aging continuum. This significantly enhances researchers’ comprehension of aging dynamics. Building on prior biomarker analyses, the hierarchical organization of aging and the focal points of each degeneration event were meticulously delineated. The establishment of aging staging relies on the subjects’ biomarker profiles and hierarchical organization, delineated by the succession of biomarker deterioration and utilizing a prior distribution informed by the sequence of biomarker decline. Leveraging the probability chain rule facilitates the management of conditional probabilities, enabling the estimation of the anticipated likelihood of subjects occupying specific aging stages.

In the ABA cohort, middle-aged individuals or middle-old-aged ABA subjects typically exhibited intermediate stages of aging, indicating a relatively slower rate of biomarker degeneration. This phenomenon likely arose from the incipient or mild nature of aging and degenerative processes in the brain during these age brackets. Conversely, elderly subjects were predominantly situated in the later stages of aging, which suggests a more conspicuous neurodegenerative progression with advancing age. This pattern resonates with typical trajectories observed in numerous neurodegenerative disorders, such as AD, where the disease severity and biomarker alterations typically intensify with age [57]. In contrast to the ABA cohort, individuals across diverse age strata in the RBA cohort demonstrated a uniform distribution across lower or intermediate stages of aging. Notably, the variations in the aging stages between different age cohorts were negligible. This observation implies that in the RBA population, age-related neurodegenerative changes may exhibit less prominence as age advances. This phenomenon could be attributed to protective mechanisms inherent within the RBA cohort, exerting influence over the pace of age-related neurodegeneration [58,59].

### 4.4. Limitation

Our research had certain limitations that warrant acknowledgment. One constraint pertained to the notable homogeneity observed within the UKB cohort, with 94.6% of participants identifying as white ethnicity. The limited ethnic diversity may restrict the applicability of our findings to more ethnically diverse populations. Moreover, the overrepresentation of white participants could limit the applicability of our results to populations characterized by diverse genetic backgrounds, lifestyles, and environmental exposures. Future studies should prioritize including more diverse cohorts to overcome this limitation. Second, the classification of ABA and RBA cohorts typically relies on brain age prediction models. In this study, we employed a more rigorous definition based on neuroimaging data from three modalities. However, many studies only utilized T1-weighted imaging data. Consequently, caution should be exercised when interpreting results, especially when cohort definitions differ. Third, the selection of biomarkers can significantly influence the interpretation of results. We selected 34 biomarkers in our study to achieve a thorough understanding of both the ABA and RBA cohorts. However, as the number of biomarkers increased, distinguishing their temporal positioning along the timeline became less apparent, posing challenges in discerning the sequence of closely related biomarker events. In future research endeavors, there is a critical need for a more comprehensive selection of biomarkers. Lastly, the DEBM provided a temporal sequence of biomarker degeneration but lacked explicit time information due to the non-linear intervals between subsequent events. Consequently, categorization into early and late events relied on comparisons with other markers over the aging trajectory. Integrating DEBM with longitudinal data and survival models could potentially provide estimates of aging progression timescales. This synergy facilitates a more nuanced understanding of the temporal dynamics underlying age-related degeneration, enabling precise delineation of the temporal aspects of the aging process.

## 5. Conclusions

Our study addressed a critical gap in the current research by investigating the temporal dynamics and neuroimaging biomarkers associated with ABA and RBA. Understanding the intricate processes of brain aging, especially the neurodegenerative mechanisms underlying these phenotypes, is crucial in neuroscience for identifying susceptibilities to cognitive decline and neurodegenerative disorders. This research contributes significantly in two main ways. First, we conducted a comprehensive examination of aging trajectories in ABA and RBA using advanced multimodal neuroimaging techniques. By integrating structural MRI, diffusion MRI, and resting-state fMRI data, we systematically explored the progression of neuroimaging biomarkers associated with ABA and RBA. This approach represents a pioneering effort to elucidate the timeline of biomarker changes in these distinct aging phenotypes, thereby advancing our understanding of their underlying mechanisms. Second, our study leveraged a large cohort of healthy volunteers (*n* = 31,621), allowing for a nuanced characterization of diverse ABA and RBA profiles. This extensive dataset provided insights into the variability and commonalities within these aging cohorts, offering a comprehensive view of how different individuals experience ABA or RBA. Ultimately, this research significantly advances our understanding of age-related cognitive decline, with the potential to reshape therapeutic strategies toward personalized interventions.

## Data Availability

The imaging datasets utilized in this study, which were derived from the UK Biobank, are accessible through the UK Biobank data access process, which is detailed at http://www.ukbiobank.ac.uk/register-apply/ (accessed on 14 January 2022). The UK Biobank’s Research Access Administration Team impartially oversees all data access inquiries from academic and commercial researchers, without bias or exclusivity. Requests undergo thorough evaluation to ensure alignment with public health research objectives, and if deemed supportive, are promptly approved. For comprehensive details regarding available data from the UK Biobank, refer to http://www.ukbiobank.ac.uk (accessed on 14 January 2022).
